# Publication practices and attitudes towards evidence-based medicine in central Asia

**DOI:** 10.1016/S2214-109X(13)70022-6

**Published:** 2013-07-24

**Authors:** Galina V Yamshchikov, George P Schmid

**Affiliations:** Centers for Disease Control and Prevention, Almaty, Kazakhstan (GPS) and Vaccine Research Center, National Institute of Allergy and Infectious Diseases, National Institutes of Health, Bethesda, MD, USA (GVY)

Evidence-based health systems are extremely important for the improvement of global health.^1^ In 1991, the five republics of central Asia (population 61 million), geopolitically strategically located and rich in natural resources, gained independence from the Soviet Union but inherited the Soviet health-care system. The Cold War limited exposure to international medical science, and reliance on Russian-oriented scientific thinking, with near-exclusive use of the Russian language in national science, created barriers to the use of and contribution to English-based sources of medical science. 20 years after independence, our experience is that these barriers persist. To assess the current contribution of central Asian scientists to medical science, we analysed the quantity and scope of medical literature from central Asia published between January, 2009, and July, 2011. To ascertain perceptions of the use of evidence-based medicine in central Asia, we also did semi-structured interviews with 85 medical scientists, medical educators, and health-care professionals from central Asia (appendix).

We identified 345 publications regarding data from central Asia and separated papers into three groups. Group A contained papers with only foreign authors—we used this category to examine publications with no influence by central Asian scientists (n=104). The remaining papers with participation of local authors were separated into two groups on the basis of the affiliation of the first author. Group B contained papers with the first author from central Asia (n=167), and group C contained papers with the first author from outside central Asia (n=74). We reasoned that projects published with the first author from central Asia were probably led by central Asian scientists, whereas the first author from outside central Asia indicated leadership by foreign scientists.

Nearly half of all publications (48%, n=167) had the first author from central Asia (5·6 publications per month). Of first-authored papers, Uzbekistan had the highest number of publications and the highest percentage of publications in English (table).

The most frequently used languages were English (260 papers) and Russian (82 papers). Papers including foreign authors (groups A and C) were mostly written in English (98/104 [94%] and 68/74 [92%], respectively), and only 95/167 (57%) of papers with the first author from central Asia (group B) were in English.

With our analysis restricted to papers with participation of a central Asian author (groups B and C), the two journals that authors published in most frequently were in Russian—*Meditsinskaia parazitologiia i parazitarnye bolezni* (*Med Parazitol*) and *Gigiena i Sanitariya (Gig Sanit).* BioMed Central was the most common English-language journal. Few journals that central Asian scientists publish in are readily accessed by Western scientists. No journals originating in central Asia are indexed in PubMed.

We categorised the main subject area for each paper on the basis of keywords or, if keywords were not available, by reviewing the abstract and making our best judgment. The subjects most often addressed were: epidemiology (n=110), public health (n=96), environmental sciences (n=28), chemistry (n=25), general medicine (n=23), genetics (n=22), pharmacology (n=15), and physics (n=9). The emphasis on health sciences was less prominent in group B publications, with pharmacology, chemistry, and physics far more common than in groups A and C. We believe this difference is consistent with the known strength of Russian basic sciences.

We examined the impact factor for all English-language journals as a proxy for quality of science. We could not evaluate the Russian-language journals by impact factor because they are not included in the Journal Citation Report. The median impact factor of articles with a central Asian author (groups B and C) was 2·53.

Of 85 interviewees, 61 were from Kazakhstan. Most interviewees (67 [79%]) were physicians and a small number (12 [14%]) characterised themselves as making health-care decisions other than at a patient level. Only 15 (18%) participants rated their English as proficient. Although 74 of 84 (88%) interviewees thought evidence-based medicine is important, most respondents (40/66 [61%]) thought that it is not used in health-care decision-making, and only 27/59 (46%) thought English-language sources were used in health-care decision-making. When asked about evidence-based medicine teaching in medical schools or postgraduate education, 34/59 (58%) and 24/54 (44%) of respondents, respectively, thought that evidence-based medicine is not provided or rarely used in the educational process. Employees of western agencies in central Asia such as UN agencies (n=7) were less likely to think evidence-based medicine was used in medical education or that English-language sources were used in health-care decision-making than were the local health-care professionals.

The results of our study indicate that countries of central Asia still have barriers to integration into world scientific processes. The disparity of scientific publications between the developing and industrialised worlds has been noted.^2^ In 2003, former UN Secretary-General Kofi Annan noted that developing nations had a population-adjusted proportion of scientists 10–30 times smaller than did developed nations and called for reduction of the resultant research gap that threatened national development.^3^ The small contribution of central Asian health scientists to world scientific literature and practice, and, conversely, the low use of knowledge from these sources, impedes health-care developments in the region and the ability to assess changes in public health of former Soviet countries.^4^

## Figures and Tables

**Table:** Publications by country of origin of first author in countries of central Asia, between January, 2009, and July, 2011

	Number of publications	Russian-language	English-language	Annualised publication number
Uzbekistan	73	23 (32%)	50 (68%)	29·2
Kazakhstan	53	25 (47%)	28 (53%)	21·2
Kyrgyzstan	24	12 (50%)	12 (50%)	9·6
Tajikistan	17	12 (71%)	5 (29%)	6·8
Turkmenistan	0	0	0	0
